# Mental health trajectories during new nurses' role transition: staged reversal of the resilience–depression and resilience–anxiety relationship

**DOI:** 10.3389/fpubh.2026.1915462

**Published:** 2026-08-06

**Authors:** Yanfei Hu, Rui Wang, Xiaolu Wei, Xuan Wang, Qian Shao, Qian Su

**Affiliations:** 1 School of Nursing, Lanzhou University, Lanzhou, Gansu, China; 2 Department of Spinal Surgery, Gansu Provincial Hospital, Lanzhou, Gansu, China

**Keywords:** new nurses, psychological resilience, depression, anxiety, longitudinal study

## Abstract

**Objective:**

This study examines the dynamic trajectories of resilience, depression, and anxiety during novice nurses' three-year role transition, with particular attention to the phase-dependent reversals in the resilience–depression and resilience–anxiety relationships, to inform temporally differentiated psychological interventions.

**Methods:**

In this prospective longitudinal cohort study, 118 newly employed nurses were recruited at baseline (T0) from a tertiary grade A hospital in Lanzhou, China (2019–2021), and 102 (86.4%) completed assessments at T0 and at the end of Years 1–3 (T1–T3). Resilience, depression, and anxiety were measured using the CD-RISC, SDS, and SAS, respectively. Data were analyzed using linear mixed-effects models, Pearson correlation with Steiger's Z-tests, group-based trajectory modeling (GBTM), and hierarchical regression.

**Results:**

Resilience increased steadily across the 3 years (64.65–81.01, *P* < 0.001). Depression followed a U-shaped trajectory, reaching its nadir at T2 (50.49) before rebounding at T3 (55.64, *P* < 0.001). In contrast to depression's U-shaped trajectory, anxiety exhibited chronic stability with only a late-stage increase from T2 to T3 (*P* = 0.004). The resilience–depression association reversed over time: negative at T0 (*r* = −0.264, *P* = 0.007), non-significant at T1–T2, and positive at T3 (*r* = 0.377, *P* < 0.001); Steiger's *Z*-test confirmed this reversal was statistically significant (*Z* = −5.720, *P* < 0.001). GBTM identified three latent trajectory classes for depression, including a low-fluctuating class (32.35%) exhibiting U-shaped recovery then rebound. Hierarchical regression revealed that explanatory power was highest at T0 → T1 and became non-significant at T2 → T3.

**Conclusion:**

The resilience–depression association shifted from negative to positive over the role transition. The depression rebound at T2 marks a critical intervention window. Anxiety demonstrated chronic stability, necessitating strategies distinct from depression-focused approaches. A staged mental health monitoring system is recommended.

## 1 Introduction

Novice nurses constitute the core reserve force of clinical care teams, and their professional competence is directly linked to patient safety and nursing quality (1). The first 3 years following entry represent a critical role transition period during which new graduates must navigate multiple challenges, including high-intensity work environments, skill acquisition, and patient-provider communication (2, 3). Research indicates that 68.41% of novice nurses experience significant transition stress during this phase (2), with 36% screening positive for depressive symptoms (4) and 54.7% reporting anxiety (5). Persistent negative affect not only elevates the risk of psychiatric morbidity and suicidal ideation but also predisposes to nursing errors and serves as a primary driver of the 35%−60% turnover rate observed within the first year (2, 6), thereby compromising care quality and amplifying health system burdens.

Psychological resilience is regarded as a core internal protective resource that enables individuals to adapt to adversity and high-pressure situations (7). Theoretically, higher resilience should buffer the impact of stress, inhibit the progression of negative affect, and sustain mental health and occupational functioning (3, 8). Elucidating the dynamic trajectories of resilience and its mechanistic links with depression and anxiety among novice nurses carries important clinical value for the development of early psychological intervention protocols.

However, several critical knowledge gaps remain unresolved in the existing literature. First, the vast majority of studies have adopted cross-sectional designs (9–11), which are inherently unable to capture the temporal dynamics of resilience, depression, and anxiety across the role transition process. Second, although a small number of studies have examined individual constructs such as depression or resilience among nurses (4, 7), these investigations have been predominantly cross-sectional in design and have not simultaneously modeled the co-development of all three constructs, leaving the interconstruct temporal dependencies largely unexplored. Third, and most fundamentally, no study to date has addressed whether the protective effect of resilience against depression and anxiety remains stable across different occupational stages or shifts as a function of time-in-role. This question is of paramount theoretical and practical importance: if resilience exerts a stable protective effect, universal resilience-enhancement programmes would be warranted; conversely, if its protective potency varies by stage, staged and precision-oriented interventions would be imperative.

Against this background, the present study employed a three-year longitudinal tracking design to conduct sustained follow-up of novice nurses, with the explicit aims to: (1) describe the independent developmental trajectories of resilience, depression, and anxiety over time; (2) reveal the phase-specific evolution of the interrelations among these three constructs; and (3) identify stage-critical influencing factors, thereby providing an empirical basis for constructing a staged, precision-oriented psychological intervention system. To our knowledge, this is the first study to simultaneously model the 3-year co-developmental trajectories of resilience, depression, and anxiety in novice nurses and to explicitly test whether the resilience–psychopathology nexus shifts as a function of occupational stage.

## 2 Materials and methods

### 2.1 Research design and participants

This study employed a prospective longitudinal cohort design, conducted at a tertiary grade A comprehensive hospital in Lanzhou, China, between 2019 and 2021.

Inclusion criteria were: (1) possession of a nurse practicing certificate; (2) newly graduated nursing students with no prior clinical work experience (excluding internships); and (3) provision of informed consent and voluntary participation.

Exclusion criteria were: (1) current diagnosis of depression, anxiety, or other psychiatric disorders; and (2) poor compliance.

Dropout criteria were: (1) voluntary withdrawal from the study; and (2) inability to complete follow-up assessments or data collection due to unforeseen circumstances (e.g., force majeure events) during the study period.

Data were collected using paper-based questionnaires administered to all newly employed nurses recruited in 2019 and thereafter. Assessments were conducted at 4 time points: the first week post-entry (baseline), and at the end of Years 1, 2, and 3. A member of the research team provided one-to-one guidance during the completion of the psychological scales for each enrolled nurse.

### 2.2 Instruments

#### 2.2.1 Novice nurse demographic questionnaire

The demographic questionnaire was developed by the research team and refined through expert consultation. It comprised basic information including gender, age, educational attainment, marital status, professional title, length of employment, sleep quality, physical exercise, monthly night shift frequency, and rotation through clinical departments.

#### 2.2.2 Connor–Davidson resilience scale (CD-RISC)

The CD-RISC is a validated instrument for assessing individuals' positive adaptation capacity and psychological resilience when facing adversity or stress (20). It comprises 25 items across three dimensions: optimism, tenacity, and self-improvement. Responses are rated on a 5-point Likert scale (0 = never, 4 = almost always), yielding a total score ranging from 0 to 100, with higher scores indicating greater resilience. In this study, the scale has good reliability and validity, and the Cronbach's alpha coefficient at each time point is 0.781–0.890.

#### 2.2.3 Self-rating depression scale (SDS)

The SDS is a widely used instrument for assessing depressive symptom severity in adults and monitoring changes in subjective depressive experiences during treatment (21). It comprises 20 items across four dimensions: depressed affect, somatic symptoms, psychomotor disturbance, and psychological disorders. Responses are rated on a 4-point Likert scale (1 = none or a little of the time, 4 = most or all of the time), yielding a raw score range of 20–80. Standard scores were derived using the conversion formula (raw total × 1.25), yielding a range of 25–100, with 53 serving as the clinical cut-off. Higher scores indicate more severe depressive symptoms. In this study, the scale has good reliability and validity, and the Cronbach's alpha coefficient at each time point is 0.758–0.828.

#### 2.2.4 Self-rating anxiety scale (SAS)

The SAS is a validated instrument for assessing anxiety symptom severity in adults and capturing subjective anxiety experiences and state changes (22). It comprises 20 items across two dimensions: somatic anxiety and psychic anxiety. Responses are rated on a 4-point Likert scale (1 = none or a little of the time, 4 = most or all of the time), yielding a raw score range of 20–80. Standard scores were derived using the conversion formula (raw total × 1.25), yielding a range of 25–100. Higher scores indicate more severe anxiety symptoms. In this study, the scale has good reliability and validity, and the Cronbach's alpha coefficient at each time point is 0.712–0.813.

### 2.3 Ethical considerations

This study was originally initiated in 2019 as a departmental quality improvement initiative, which did not require formal ethics committee approval at that time; internal administrative approval was obtained from Gansu Provincial People's Hospital. All participants provided written informed consent at baseline. When the decision was made to publish the findings as academic research, a retrospective ethics application was submitted to the Medical Ethics Committee, which granted formal approval in 2022 (Approval No. 2022-001). The study was conducted in accordance with the Declaration of Helsinki.

### 2.4 Statistical analysis

Data were entered in duplicate using EpiData 3.1 (The EpiData Association, Odense, Denmark) and cross-verified. Analyses were performed using SPSS 26.0 and (IBM Corporation, Armonk, New York, USA) Mplus 8.3 (Muthén & Muthén, Los Angeles, California, USA), with two-tailed *P* < 0.05 indicating statistical significance.

Linear mixed-effects models (LMM) with robust standard errors were fitted to examine the main effect of time on each construct, including time, gender, and age as fixed effects with random intercepts for participants. Partial eta squared (η^2^) quantified effect size (≥0.14 = large, 0.06–0.14 = medium, <0.06 = small).

Pearson correlation analyses were conducted at each assessment point. Steiger's *Z*-tests were used to compare the magnitude of correlations between T0 and T3.

Group-based trajectory modeling (GBTM) was conducted using Mplus 8.3. Censored normal models with 2–4 classes were fitted for each construct; model selection followed BIC, entropy ≥ 0.70, LMR-LRT *P* < 0.05, and minimum class size ≥ 5%.

Hierarchical regression was employed to identify predictors of change scores at each stage, with predictors entered in three blocks: demographic characteristics, work-related factors, and psychosocial factors. Models that were non-significant overall (*P* > 0.05) were not reported.

## 3 Results

**Figure 1:**
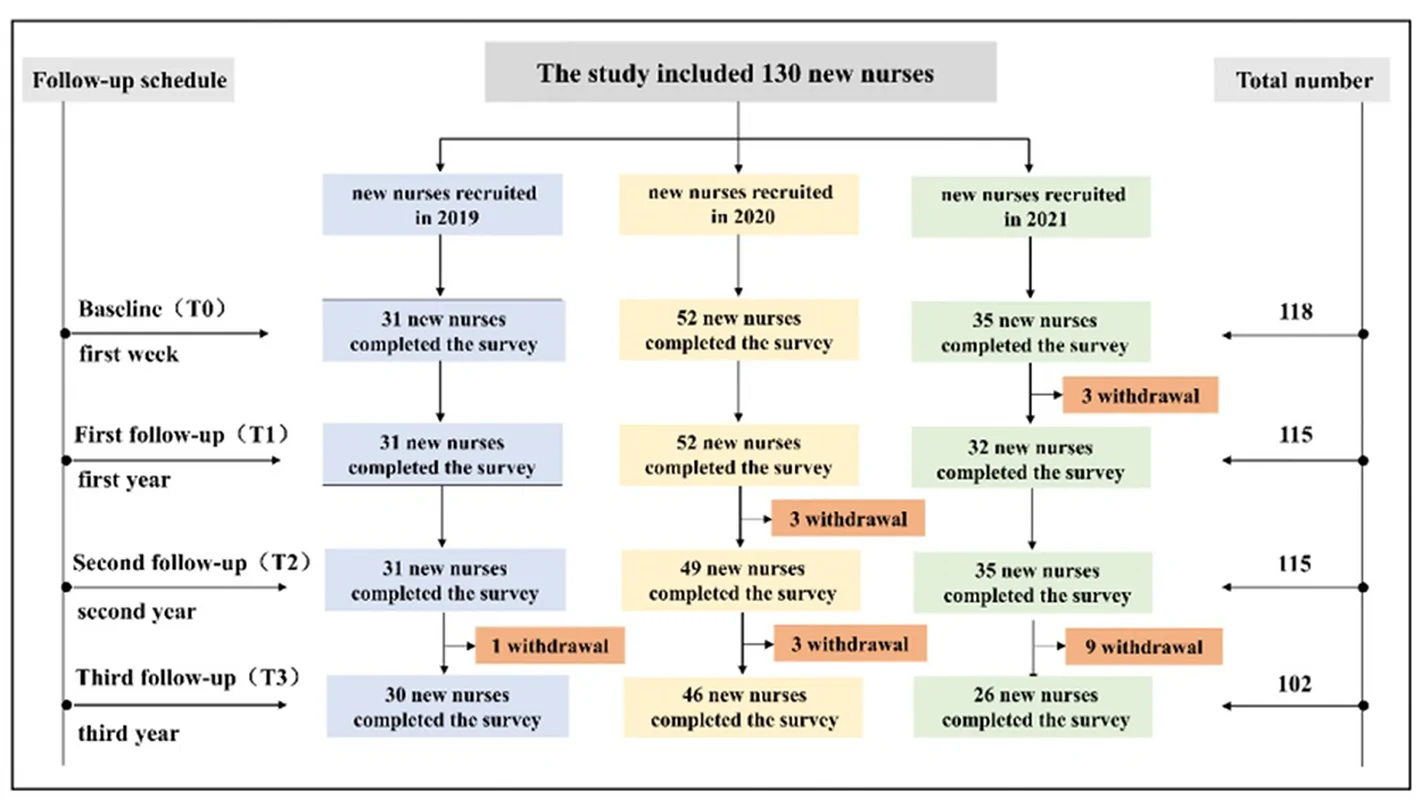
Longitudinal study object into the flow chart.

A total of 130 new nurses were initially enrolled at baseline, with 118 completing the T0 survey. Over the three-year follow-up, 16 participants withdrew from the study: 3 withdrew between T0 and T1, 3 withdrew between T1 and T2, and 10 withdrew between T2 and T3 (Figure 1). All withdrawals were due to uncontrollable factors such as pregnancy, resignation, or job transfer to non-participating hospitals. The final analytical sample comprised 102 participants with complete data across all four assessments. Demographic characteristics are presented in Table 1.

**Table 1:** General information of novice nurses (*n* = 102).

Variable	T0		T1		T2		T3	
	*n*	%	*n*	%	*n*	%	*n*	%
Gender								
Man	14	13.73	14	13.73	14	13.73	14	13.73
Woman	88	86.27	88	86.27	88	86.27	88	86.27
Age, y								
20–25	90	88.24	72	70.59	59	57.84	40	39.22
26–30	10	9.8	30	29.41	43	42.16	61	59.8
>30	2	1.96	0	0	0	0	1	0.98
Ethnicity								
Han	96	94.12	96	94.12	96	94.12	96	94.12
Minority	6	5.88	6	5.88	6	5.88	6	5.88
Degree of education								
Bachelor	95	93.14	95	93.14	95	93.14	95	93.14
Master	7	6.86	7	6.86	7	6.86	7	6.86
Single child								
No	90	88.24	90	88.24	90	88.24	90	88.24
Yes	12	11.76	12	11.76	12	11.76	12	11.76
Marital status								
Single	102	100	102	100	98	96.08	75	73.53
Married	0	0	0	0	4	3.92	27	26.47
Title								
Nurse	88	86.27	100	98.04	62	60.78	24	23.53
Primary nurse	14	13.73	2	1.96	37	36.27	76	74.51
Nurse-in-charge	0	0	0	0	3	2.94	2	1.96
Year of employment, y								
2019	25	24.51	25	24.51	25	24.51	25	24.51
2020	43	42.16	43	42.16	43	42.16	43	42.16
2021	34	33.33	34	33.33	34	33.33	34	33.33
Number of night shifts per month								
0	102	100	44	43.14	14	13.73	12	11.76
1–5	0	0	40	39.22	50	49.02	49	48.04
6–10	0	0	18	17.65	35	34.31	40	39.22
>10	0	0	0	0	3	2.94	1	0.98
Sleep situation								
Fall asleep quickly	85	83.33	77	75.49	70	68.63	64	62.75
Difficult to fall asleep	12	11.76	18	17.65	24	23.53	29	28.43
Toss and turn restlessly	5	4.9	7	6.86	8	7.84	9	8.82
Sleep quality								
Not easy to wake up	32	31.37	27	26.47	29	28.43	43	42.16
Often have nightmares	7	6.86	12	11.76	12	11.76	19	18.63
Easy to wake up	63	61.76	63	61.76	61	59.8	40	39.22
Sports activities								
Hardly attend	21	20.59	15	14.71	36	35.29	52	50.98
Take part occasionally	56	54.9	78	76.47	57	55.88	34	33.33
Attend every day	25	24.51	9	8.82	9	8.82	16	15.69
Rotation department								
Internal medicine	14	13.73	31	30.39	22	21.57	33	32.35
Surgery	27	26.47	26	25.49	36	35.29	32	31.37
emergency department	7	6.86	9	8.82	6	5.88	4	3.92
ICU	7	6.86	11	10.78	7	6.86	9	8.82
Gynecology and pediatrics	4	3.92	6	5.88	8	7.84	3	2.94
Operating room	29	28.43	9	8.82	17	16.67	18	17.65
Other departments	14	13.73	10	9.8	6	5.88	3	2.94

Data are presented as frequency and percentage (*n*, %).

### 3.1 Longitudinal trajectories of resilience, depression, and anxiety

**Figure 2:**
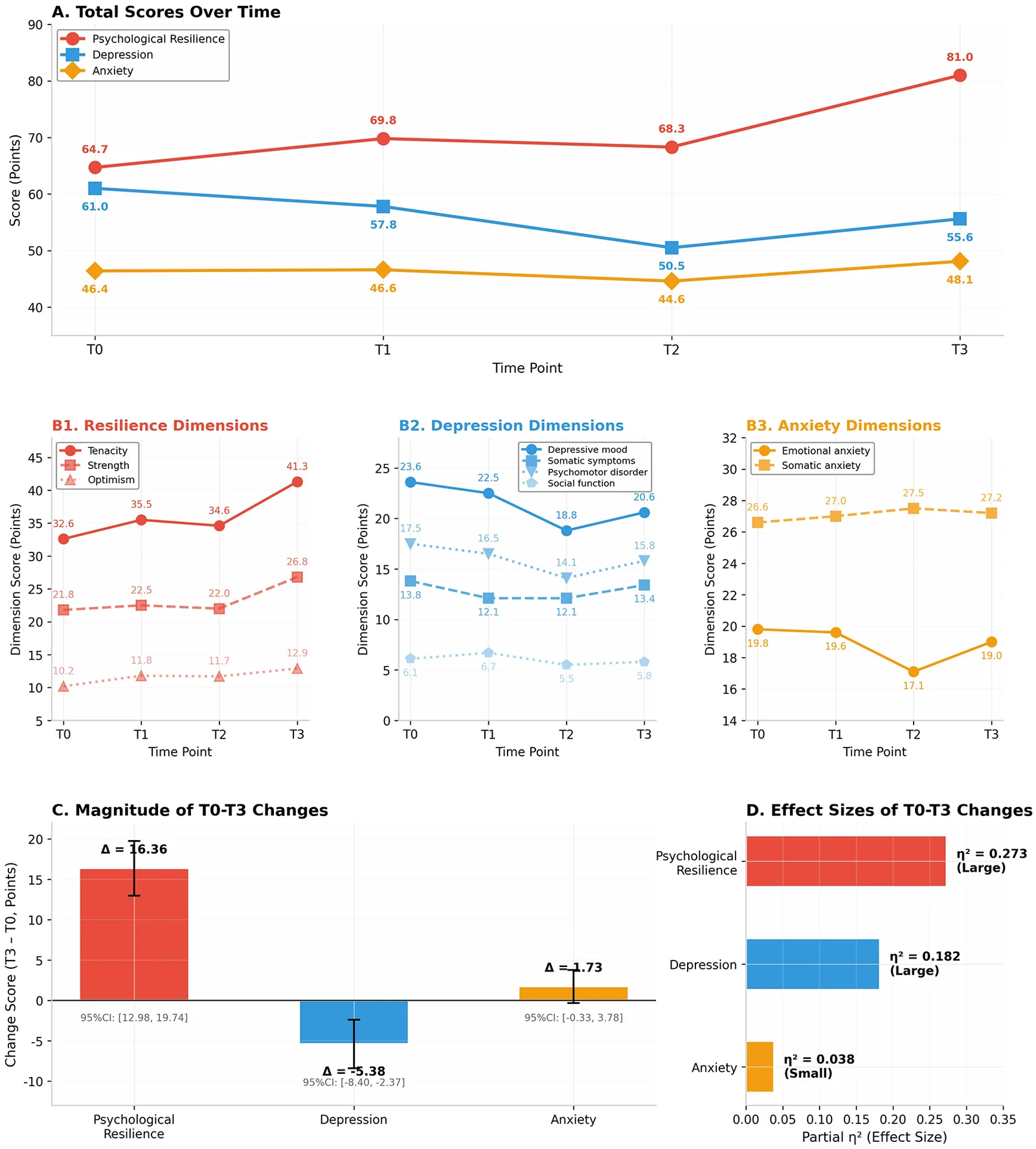
Longitudinal changes in psychological resilience, depression, and anxiety among novice nurses. **(A)** Total score trajectories. **(B)** Dimension-level trajectories (**B1**: resilience, **B2**: depression, **B3**: anxiety). **(C)** Magnitude of T0–T3 changes with 95% confidence intervals. **(D)** *Partial* η^2^ effect sizes. Effect size benchmarks: Small (η^2^ ≥ 0.01), Medium (η^2^ ≥ 0.06), Large (η^2^ ≥ 0.14).

LMM analyses (Table 2, Figure 2) revealed significant main effects of time for all three constructs. Resilience increased steadily from T0 (64.65 ± 10.19) to T3 (81.01 ± 17.86; χ^2^ = 52.754, *P* < 0.001, η^2^ = 0.25), with all subscales following an identical upward pattern. Depression displayed a U-shaped trajectory: declining from T0 (61.02 ± 10.19) to a nadir at T2 (50.49 ± 12.12), before rebounding to 55.64 ± 9.99 at T3 (χ^2^ = 51.741, *P* < 0.001, η^2^ = 0.18). Pairwise comparisons (Table 3, Figure 2) confirmed significant T2 → T3 rebound (*Cohen's d* = 0.36, *P* = 0.003). Anxiety showed minimal change (χ^2^ = 17.100, *P* < 0.001), with only the T2 → T3 comparison reaching significance (*d* = 0.35, *P* = 0.004); the total change from T0 to T3 was 1.73 points (95% CI: −0.33 to 3.78, P = 0.102).

**Table 2:** Longitudinal trajectories of resilience, depression, and anxiety among novice nurses.

Variable	T0	T1	T2	T3	*χ^2^*	*df*	*P*
Psychological resilience total score	64.65 ± 10.19	69.80 ± 14.91	68.26 ± 16.45	81.01 ± 17.86	52.754	3	<0.001^***^
Psychological resilience mean score	2.59 ± 0.41	2.79 ± 0.60	2.73 ± 0.66	3.24 ± 0.71	52.754	3	<0.001^***^
Tenacity	2.51 ± 0.49	2.73 ± 0.64	2.64 ± 0.70	3.17 ± 0.76	46.23	3	<0.001^***^
Strength	2.77 ± 0.42	2.97 ± 0.62	2.89 ± 0.68	3.35 ± 0.77	37.15	3	<0.001^***^
Optimism	2.47 ± 0.49	2.65 ± 0.62	2.69 ± 0.67	3.23 ± 0.72	55.89	3	<0.001^***^
Total score of self-rating depression	61.02 ± 10.19	57.81 ± 6.00	50.49 ± 12.12	55.64 ± 9.99	51.741	3	<0.001^***^
Total average score of self-rating depression	3.05 ± 0.51	2.89 ± 0.30	2.52 ± 0.61	2.78 ± 0.50	51.741	3	<0.001^***^
Depressive mood	2.27 ± 0.78	2.02 ± 0.55	2.01 ± 0.57	2.23 ± 0.67	8.52	3	0.036^*^
Somatic symptoms	2.79 ± 0.52	2.75 ± 0.45	2.34 ± 0.68	2.68 ± 0.64	32.45	3	<0.001^***^
Psychomotor disorder	3.91 ± 0.81	3.76 ± 0.74	3.03 ± 1.02	3.35 ± 0.85	53.89	3	<0.001^***^
Social function	3.49 ± 0.96	3.45 ± 0.81	3.15 ± 1.08	3.31 ± 0.97	5.89	3	0.117
Total score of self-rating anxiety	46.41 ± 6.76	46.62 ± 7.77	44.62 ± 9.84	48.14 ± 10.84	17.100	3	<0.001^***^
Total average score of self-rating anxiety	2.32 ± 0.34	2.33 ± 0.39	2.23 ± 0.49	2.41 ± 0.54	17.100	3	<0.001^***^
Emotional anxiety	3.35 ± 0.57	3.32 ± 0.62	2.94 ± 0.76	3.16 ± 0.70	20.12	3	<0.001^***^
Somatic anxiety	1.87 ± 0.36	1.91 ± 0.55	1.93 ± 0.53	2.08 ± 0.59	12.34	3	0.006^**^

Data are presented as mean ± standard deviation. χ^2^ values represent the likelihood ratio test for the main effect of time from linear mixed-effects models. All LMMs included time, gender, and age as fixed effects, with random intercepts for participants. Robust standard errors were used to account for residual non-normality. ^*^P < 0.05, ^**^P < 0.01, ^***^P < 0.001.

**Table 3:** *Bonferroni-corrected* pairwise comparisons of depression, anxiety, and psychological resilience across 4 time points (M ± SD).

Variable	Comparison	*M_*diff*_*	*SE*	95% CI [LL, UL]	*t*	df	*p*	*p_*adj*_*	*Cohen's d*	Sig.
Resilience	T0 vs T1	5.16	1.42	(2.38, 7.93)	3.64	101	<0.001	0.003	0.36	^**^
Resilience	T0 vs T2	3.62	1.69	(0.31, 6.93)	2.14	101	0.035	0.207	0.21	ns
Resilience	T0 vs T3	16.36	1.72	(12.98, 19.74)	9.49	101	<0.001	<0.001	0.94	^***^
Resilience	T1 vs T2	−1.54	1.23	(−3.94, 0.86)	−1.26	101	0.212	1.00	−0.12	ns
Resilience	T1 vs T3	11.21	1.77	(7.73, 14.68)	6.31	101	<0.001	<0.001	0.63	^***^
Resilience	T2 vs T3	12.75	1.81	(9.20, 16.29)	7.05	101	<0.001	<0.001	0.70	^***^
Depression	T0 vs T1	−3.22	1.02	(−5.22, −1.21)	−3.14	101	0.002	0.013	−0.31	^*^
Depression	T0 vs T2	−10.53	1.44	(−13.35, −7.71)	−7.32	101	<0.001	<0.001	−0.72	^***^
Depression	T0 vs T3	−5.38	1.54	(−8.40, −2.37)	−3.50	101	<0.001	0.004	−0.35	^**^
Depression	T1 vs T2	−7.32	1.33	(−9.92, −4.71)	−5.51	101	<0.001	<0.001	−0.55	^***^
Depression	T1 vs T3	−2.17	1.09	(−4.30, −0.04)	−2.00	101	0.048	0.29	−0.20	ns
Depression	T2 vs T3	5.15	1.43	(2.35, 7.94)	3.61	101	<0.001	0.003	0.36	^**^
Anxiety	T0 vs T1	0.21	0.91	(−1.57, 1.98)	0.23	101	0.818	1.00	0.02	ns
Anxiety	T0 vs T2	−1.79	0.93	(−3.62, 0.04)	−1.92	101	0.058	0.35	−0.19	ns
Anxiety	T0 vs T3	1.73	1.05	(−0.33, 3.78)	1.65	101	0.102	0.613	0.16	ns
Anxiety	T1 vs T2	−2.00	1.04	(−4.03, 0.04)	−1.92	101	0.057	0.344	−0.19	ns
Anxiety	T1 vs T3	1.52	1.14	(−0.72, 3.75)	1.33	101	0.186	1.00	0.13	ns
Anxiety	T2 vs T3	3.52	1.00	(1.56, 5.48)	3.52	101	<0.001	0.004	0.35	^**^

*M*__*diff*__, mean difference (later time point minus earlier time point); *SE*, standard error; CI, confidence interval; LL, lower limit; UL, upper limit; *p*, uncorrected *p*-value; *p*_*adj*_, *Bonferroni-adjusted p*-value (α = 0.0083, 0.05/6 comparisons); *Cohen's d*, paired-samples effect size. ^*^p < 0.05, ^**^p < 0.01, ^***^p < 0.001 (*Bonferroni-corrected*). ns, not significant.

### 3.2 Phase-specific evolution of interconstruct relations

Pearson correlations (Table 4) revealed marked shifts across assessment points. At T0, resilience was negatively correlated with both depression (*r* = −0.264, *P* = 0.007) and anxiety (*r* = −0.215, *P* = 0.030). At T1–T2, resilience showed no significant association with either construct. At T3, a critical reversal occurred: resilience became positively correlated with depression (*r* = 0.377, *P* < 0.001), whilst its association with anxiety remained non-significant (*r* = 0.022, *P* = 0.824).

**Table 4:** Correlations among resilience, depression, and anxiety in novice nurses at T0–T3.

Time point	Depression–anxiety		Depression–resilience		Anxiety–resilience	
	*r*	*P*	*r*	*P*	*r*	*P*
T0	0.211	0.033^*^	−0.264	0.007^**^	−0.215	0.030^*^
T1	0.354	<0.001^***^	0.206	0.038^*^	−0.169	0.090
T2	0.546	<0.001^***^	−0.007	0.948	−0.189	0.058
T3	0.568	<0.001^***^	0.377	<0.001^***^	0.022	0.824

^*^p < 0.05, ^**^p < 0.01, ^***^p < 0.001.

Steiger's *Z*-tests (Table 5) confirmed that these changes were statistically significant: the resilience–depression correlation shifted from negative at T0 to positive at T3 (*Z* = −5.720, *P* < 0.001), as did the depression–anxiety correlation, which strengthened over time (*Z* = −3.565, *P* < 0.001). The resilience–anxiety correlation also differed significantly between time points (*Z* = −2.059, *P* = 0.039).

**Table 5:** Comparison of correlation coefficients across time points.

Pair	T0(*r*)	T3(*r*)	Steiger's *Z*-test	
			*Z*	*p*
Resilience–Depression	−0.264	0.377	−5.720	<0.001^***^
Resilience–Anxiety	−0.215	0.022	−2.059	0.039^*^
Depression–Anxiety	0.211	0.568	−3.565	<0.001^***^

^*^p < 0.05, ^***^p < 0.001. Negative *Z* values indicate the T0 correlation was significantly lower (more negative) than the T3 correlation; positive *Z* values indicate the opposite.

### 3.3 Heterogeneity of individual change trajectories

**Figure 3:**
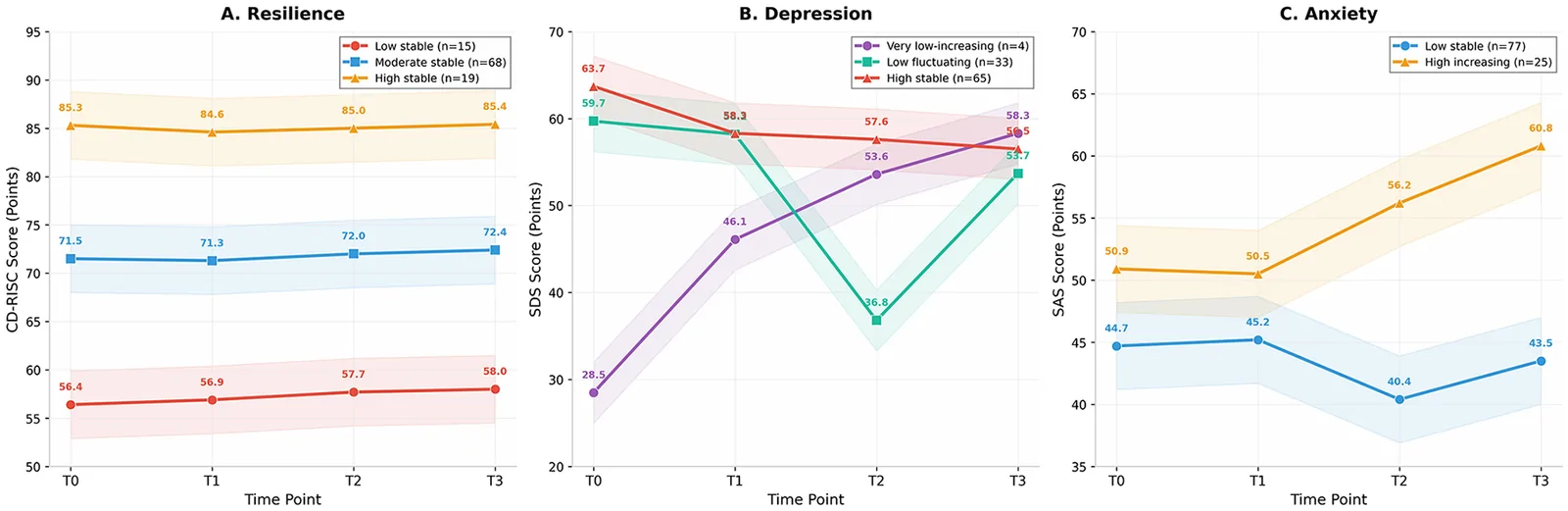
Group-based trajectory model (GBTM) estimated trajectories of resilience, depression, and anxiety across 4 time points (T0–T3). **(A)** Resilience, **(B)** Depression, **(C)** Anxiety. Solid lines represent estimated group means, shaded areas indicate 95% confidence intervals, and numeric values above each data point denote the estimated mean score at each time point.

GBTM identified distinct latent trajectory classes for each construct (Tables 6, 7). For resilience, a 3-class linear model was selected (BIC = 3,027.127, entropy = 0.844): low-stable (14.71%), moderate-stable (66.67%), and high-stable (18.63%). For depression, a 3-class quadratic model was selected (BIC = 3,014.883, entropy = 0.844): very low to increasing (3.92%), U-shaped (32.35%), and high depression stable (63.73%). The U-shaped class exhibited the characteristic U-shaped pattern (Figure 3). For anxiety, a 2-class linear model was selected (BIC = 2,889.237, entropy = 0.836): low-stable (75.49%) and high-increasing (24.51%).

**Table 6:** Model fit indices for group-based trajectory models of resilience, depression, and anxiety.

Scale	K	Model Type	Polynomial	AIC	BIC	Entropy	LMR-LRT	BLRT	Min Class %	Selected
Resilience	2	Censored Normal	Linear	3,014.883	3,038.133	0.722	<0.001^***^	<0.001^***^	13.73%	
Resilience	3	Censored Normal	Linear	2,991.752	3,027.127	0.844	0.060	<0.001^***^	13.73%	Yes
Resilience	4	Censored Normal	Linear	2,975.508	3,014.883	0.844	0.220	<0.001^***^	3.92%	
Depression	2	Censored Normal	Linear	3,003.12	3,029.370	0.709	0.060	<0.001^***^	32.35%	
Depression	3	Censored Normal	Quadratic	2,975.508	3,014.883	0.844	0.220	<0.001^***^	3.92%	Yes
Depression	4	Censored Normal	Quadratic	2,947.848	3,000.347	0.866	0.197	<0.001^***^	3.92%	
Anxiety	2	Censored Normal	Linear	2,862.987	2,889.237	0.836	<0.001^***^	<0.001^***^	24.51%	Yes
Anxiety	3	Censored Normal	Linear	2,860.138	2,899.512	0.817	0.283	0.333	9.80%	
Anxiety	4	Censored Normal	Linear	2,856.092	2,908.592	0.859	0.632	0.177	2.94%	

K, number of trajectory classes; AIC, Akaike Information Criterion; BIC, Bayesian Information Criterion; LMR-LRT, Lo-Mendell-Rubin likelihood ratio test; BLRT, bootstrapped likelihood ratio test. Censored normal models were fitted with linear or quadratic polynomial functions. The selected model for each scale (indicated by “Yes”) was determined based on the lowest BIC, entropy ≥ 0.70, LMR-LRT *p* < 0.05, and a minimum class size ≥ 5%. ^***^p < 0.001.

**Table 7:** Estimated means, standard errors, and 95% confidence intervals for GBTM trajectory classes across 4 time points.

Scale	Class	*n* (%)	T0	T1	T2	T3
			*M* (*SE*) [95% CI]	*M* (*SE*) [95% CI]	*M* (*SE*) [95% CI]	*M* (*SE*) [95% CI]
Resilience (3-class)	Low resilience stable	15 (14.71%)	56.40 (1.48) [53.23, 59.57]	56.90 (1.49) [53.70, 60.10]	57.70 (1.48) [54.53, 60.87]	58.00 (1.49) [54.80, 61.20]
Resilience (3-class)	Moderate resilience stable	68 (66.67%)	71.50 (0.79) [69.92, 73.08]	71.30 (0.79) [69.72, 72.88]	72.00 (0.79) [70.42, 73.58]	72.40 (0.79) [70.82, 73.98]
Resilience (3-class)	High resilience stable	19 (18.63%)	85.30 (0.92) [83.37, 87.23]	84.60 (0.92) [82.67, 86.53]	85.00 (0.92) [83.07, 86.93]	85.40 (0.92) [83.47, 87.33]
Depression (3-class)	Very low to increasing	4 (3.92%)	28.50 (5.42) [11.25, 45.75]	46.10 (5.43) [28.82, 63.38]	53.60 (5.43) [36.32, 70.88]	58.30 (5.43) [41.02, 75.58]
Depression (3-class)	U-shaped	33 (32.35%)	59.70 (1.92) [55.79, 63.61]	58.20 (1.92) [54.29, 62.11]	36.80 (1.92) [32.89, 40.71]	53.70 (1.92) [49.79, 57.61]
Depression (3-class)	High depression stable	65 (63.73%)	63.70 (1.37) [60.96, 66.44]	58.30 (1.37) [55.56, 61.04]	57.60 (1.37) [54.86, 60.34]	56.50 (1.37) [53.76, 59.24]
Anxiety (2-class)	Low anxiety stable	77 (75.49%)	44.70 (0.72) [43.27, 46.13]	45.20 (0.72) [43.77, 46.63]	40.40 (0.72) [38.97, 41.83]	43.50 (0.72) [42.07, 44.93]
Anxiety (2-class)	High anxiety increasing	25 (24.51%)	50.90 (1.26) [48.30, 53.50]	50.50 (1.26) [47.90, 53.10]	56.20 (1.26) [53.60, 58.80]	60.80 (1.26) [58.20, 63.40]

GBTM, group-based trajectory model; *M*, mean; *SE*, standard error; CI, confidence interval.

Within depression trajectory groups, baseline depression scores differed significantly (*H* = 42.02, *P* < 0.001): the improved group had the highest baseline (67.42 ± 6.51), whilst the deteriorated group had the lowest (51.18 ± 11.28).

### 3.4 Hierarchical effects of predictors across stages

Hierarchical regression (Table 8) revealed stage-specific patterns. At T0 → T1, psychosocial factors demonstrated substantial explanatory power for depression change (adjusted *R*^2^ increased to 0.685) and anxiety change (adjusted *R*^2^ = 0.351), with baseline scores as significant negative predictors. Resilience change was primarily explained by demographic factors (adjusted *R*^2^ = 0.346). At T1 → T2, only the anxiety model remained significant (adjusted *R*^2^ = 0.197). At T2 → T3, explanatory power attenuated substantially: only the depression model retained marginal significance (adjusted *R*^2^ = 0.010, *P* = 0.041), with baseline depression as the sole predictor, whilst resilience and anxiety models became non-significant.

**Table 8:** Hierarchical regression models for longitudinal changes in resilience, depression, and anxiety among novice nurses.

Outcome	Model 1		Model 2		Model 3		Significant predictors [β(*P*)]
	Adjusted *R^2^*	Δ*R^2^*(1)	Adjusted *R^2^*	Δ*R^2^*(2)	Adjusted *R^2^*	Δ*R^2^*(3)	
Resilience change (T0 → T1)	0.265^***^	0.301^***^	0.257	0.022	0.346^***^	0.088^***^	Age (6.14, <0.001^***^); Degree of education (−21.15, 0.020^*^); Resilience-T0 (−0.43, <0.001^***^)
Resilience change (T1 → T2)	0.071	0.117	0.037	0.006	0.05	0.022	Gender (11.81, 0.008^**^); Ethnicity (11.53, 0.030^*^)
Resilience change (T2 → T3)	0.034	0.082	0.022	0.027	0.019	0.007	None
Depression change (T0 → T1)	0.069	0.115	0.114	0.078	0.685^***^	0.523^***^	Degree of education (10.56, 0.022^*^); Depression-T0 (−0.82, <0.001^***^)
Depression change (T1 → T2)	−0.024	0.027	−0.014	0.05	−0.024	0.001	None
Depression change (T2 → T3)	−0.014	0.036	−0.025	0.03	0.010^*^	0.042^*^	Depression-T0 (−0.32, 0.041^*^)
Anxiety change (T0 → T1)	0.004	0.054	0.02	0.054	0.351^***^	0.314^***^	Degree of education (13.27, 0.024^*^); Anxiety-T0 (−0.76, <0.001^***^)
Anxiety change (T1 → T2)	0.002	0.051	0.027	0.063	0.197^***^	0.171^***^	Sports activities (3.29, 0.029^*^); Resilience-T0 (−0.36, <0.001^***^)
Anxiety change (T2 → T3)	−0.016	0.034	−0.042	0.017	−0.048	0.015	None

Δ*R*^2^ represents the incremental explanatory power of the current model relative to the preceding model. β denotes unstandardised regression coefficients. ^*^p < 0.05, ^**^p < 0.01, ^***^p < 0.001.

## 4 Discussion

### 4.1 The staged reversal of the resilience–depression nexus

The central finding of this study is that the association between resilience and depression shifted from negative to positive over the three-year role transition—a pattern formally confirmed by Steiger's *Z*-tests. This challenges the conventional conceptualization of resilience as a stable protective factor (12) and aligns with emerging evidence that resilience–psychopathology relations are context-dependent (13).

We offer three speculative mechanisms for this reversal. First, the qualitative shift in role demands at T3—from structured training to independent practice—may transform resilience from a stress-buffer into a risk factor. Nurses with high self-reported resilience may overestimate their capabilities and undertake responsibilities beyond their competence, or refrain from seeking help to avoid displaying vulnerability (14). This aligns with Labrague and De Los Santos' (15) finding that transition shock peaks during the shift from dependent to independent practice. Second, the CD-RISC primarily captures self-reported resilience rather than actual coping capacity (16); high scorers may simultaneously exhibit elevated perfectionism, which functions as a protective factor under low-pressure conditions but becomes maladaptive under high-accountability stress (17). Third, highly resilient individuals may be perceived as “not needing help” and consequently overlooked by clinical management (18), leading to concealed stress accumulation. These mechanisms are not mutually exclusive and require empirical validation in future studies.

This finding carries direct clinical implications: resilience scores alone should not guide mental health triage. The transition to independent practice warrants proactive outreach to nurses scoring high on resilience measures.

### 4.2 Depression U-shaped trajectory: T2 as a critical intervention window

The U-shaped depression trajectory—with rebound at T2 → T3—represents a novel finding in novice nurse longitudinal research. The initial decline (T0 → T2) likely reflects the structured support of standardized training, whilst the rebound coincides with the abrupt withdrawal of this support upon commencement of independent practice (2, 15).

The GBTM-identified low-fluctuating class (32.35%) is particularly instructive: these nurses improved to T2 then deteriorated, suggesting that training-phase improvement may mask underlying vulnerability. Relying solely on T2 depression scores to infer adaptation status would miss this inflection point—a phenomenon with direct implications for transition support planning.

### 4.3 Anxiety chronicity: a distinct pathological process

Unlike depression, anxiety showed minimal change over 3 years, exhibiting chronic maintenance. This dissociation suggests distinct pathological mechanisms: depression may represent a reactive process amenable to environmental modification (19), whilst anxiety may reflect trait-like anticipatory worry resistant to situational change (17). The GBTM high-increasing anxiety class (24.51%) further underscores the need for anxiety-specific interventions—transposing depression-focused strategies onto anxiety may be ineffective.

### 4.4 Declining predictive power

The progressive attenuation of regression model explanatory power—from substantial at T0 → T1 to non-significant at T2 → T3—indicates that baseline psychological states and demographic characteristics possess strong predictive value only for short-term changes. This finding challenges the “single-point screening approach” approach to nurse mental health monitoring (6) and supports the implementation of dynamic, continuous assessment systems.

### 4.5 Limitations

This study has several limitations. First, the single-center design limits generalisability; multi-center replication is needed. Second, unmeasured confounders including organizational support and job satisfaction were not collected. Third, the absence of a priori power calculation is acknowledged, though post hoc analyses indicate adequate power for the primary analyses.

## 5 Conclusion

This 3-year longitudinal study demonstrates that the resilience–depression association reverses from negative to positive during novice nurses' role transition. The depression rebound at T2 marks a critical intervention window. Anxiety demonstrates chronic stability, necessitating strategies distinct from depression-focused approaches. Predictive models become ineffective during independent practice, indicating that dynamic monitoring systems should replace reliance on entry screening. A staged mental health support framework, with proactive outreach to high-resilience nurses at the T2 → T3 transition, is recommended.

## Data Availability

The raw data supporting the conclusions of this article will be made available by the authors, without undue reservation.

## References

[1] Kukkonen P Koskinen S Fuster-Linares P Istomina N Leino-Kilpi H Löyttyniemi E . The professional competence of newly graduated nurses in the transition phase as assessed by nurse managers: a descriptive cross-sectional multi-national study. J Res Nurs. (2025) 30:210–29. doi: 10.1177/17449871241311543 40099198 PMC11910741

[2] Su Q Wu Y Yun B Zhang H She D. Han, L. The mediating effect of clinical teching behavior on transition shock career identity among new nurses: a cross-sectional study. Nurse Educ Today. (2023) 125:105780. doi: 10.1016/j.nedt.2023.105780 36963229

[3] See ECW Koh SSL Baladram S Shorey S. Role transition of newly graduated nurses from nursing students to registered nurses: a qualitative systematic review. Nurs Educ. Today (2023) 121. doi: 10.1016/j.nedt.2022.105702 36577288

[4] Jose S Cyriac MC Dhapani M Mehra A Sharma N. Mental health outcomes of perceived stress, anxiety, fear insomnia, the resilience among frontline nurses caring for critical COVID-19 patients in intensive care units. Indian J Crit Care Med. (2022) 26:174–8. doi: 10.5005/jp-journals-10071-24119 35712741 PMC8857709

[5] Ghahramani S Kasraei H Hayati R Tabrizi R Marzaleh MA. Health care workers' mental health in the face of COVID-19: a systematic review meta-analysis. Int J Psychiatr Clin Prac. (2023) 27:208–17. doi: 10.1080/13651501.2022.2101927 35875844

[6] Wu YH. A longitudinal study on occupational stress and mental health among new nurses (Master's thesis). Lanzhou University (2023). doi: 10.27204/d.cnki.glzhu.2023.002108

[7] Liu X Luo L Ye Z Zhou J Bai Q. Research progress on resilience of HIV/AIDS patients. Chin J Hum Sex. (2025) 34:131–35. doi: 10.3969/j.issn.1672-1993.2025.04.029

[8] Ji X Wang S Feng H Ma J Zhao H Li Q. Study on the current situation and influencing factors of new nurses' workplace adaptability. J Nurs Adm. (2022) 22:759–63. doi: 10.3969/j.issn.1671-315x.2022.10.013

[9] Norman PI Griffiths PP. Introducing the international journal of nursing studies—advances. Int J Nurs Stud. (2022) 127. doi: 10.1016/j.ijnurstu.2022.104177 35093256

[10] Zheng Q Su X Nie A Li J. The mediating effect between the transition shock of new nurses and job readiness in psychological resilience. Health Vocat Educ. (2025) 43:135–40. doi: 10.20037/j.issn.1671-1246.2025.03.35

[11] Catarelli B Nobles P Aull M Yi F. Evaluating burnout resiliency in new graduate nurses. JONA: J Nurs Adm. (2023) 53:259–265. doi: 10.1097/NNA.0000000000001279 37098865

[12] Rutter M. Resilience: some conceptual considerations. J Adolesc Health (1993) 14:626–631:690–696. doi: 10.1016/1054-139X(93)90196-V 8130234

[13] Baminiwatta A Fernando R Gadambanathan T Jiyatha F Maryam KH Premaratne T. et al. The buffering role of resilience on burnout, depression, anxiety, stress among healthcare workers in Sri Lanka. Discov Psychol. (2025) 5. doi: 10.1007/s44202-025-00345-4

[14] Guo J Meng Y Li Y. The mediating and moderating effects of resilience on transition shock and depersonalization of new nurses. Chin J Health Psychol. (2021) 29:236–40. doi: 10.13342/j.cnki.cjhp.2021.02.016

[15] Labrague LJ De Los Santos JAA. Transition shock newly graduated nurses' job outcomes select patient outcomes: a cross-sectional study. J Nurs Manag. (2020) 28:1070–1079. doi: 10.1111/jonm.13033 32315478

[16] Connor KM Davidson JRT. Development of a new resilience scale: the Connor-Davidson resilience scale (CD-RISC). Depress Anxiety. (2003) 18:76–82. doi: 10.1002/da.10113 12964174

[17] Hosseini Z Homayuni A. Personality occupational correlates of anxiety depression in nurses: the contribution of role conflict, core self-evaluations, negative affect bullying. BMC Psychol. (2022) 10:215. doi: 10.1186/s40359-022-00921-6 36088398 PMC9463792

[18] Kolutek R Erkutlu H Chafra J. Workplace violence nurses' psychological well-being: the mediating role of burnout the moderating role of psychological resilience. Arch Psychiatr Nurs. (2024) 53:177–183. doi: 10.1016/j.apnu.2024.10.015 39615933

[19] Zhou Y Wang S Liu M Gan G Qin N Luo X . The role of sleep quality perceived stress on depressive symptoms among tertiary hospital nurses: a cross-sectional study. BMC Psychiatr. (2023) 23:416. doi: 10.1186/s12888-023-04936-0 37308915 PMC10258928

[20] Li XL Zhang D Yang RH Tian XQ Huang G Yue X . Influence of psychological intervention based on the three-in-one concept of “cognition, behavior and humanism” on psychological resilience and post-traumatic growth in patients with vascular malformation surgery. Chin J Health Psychol. (2025) 33:1098–1103. doi: 10.13342/j.cnki.cjhp.2025.07.027

[21] Hai B Xu Z Li Y. Effect of psychological intervention on mental health and quality of life of patients with shoulder pain. Chin J Health Psychol. (2025) 33:382–86. doi: 10.13342/j.cnki.cjhp.2025.03.012

[22] Yu S Wang J Liu W Zhu X Mao X Yu J . Investigation and analysis of the mental state of healthy volunteers in the first phase of clinical research of new drugs. Chin J Clin Pharmacol. (2025) 41:1310–14. doi: 10.13699/j.cnki.1001-6821.2025.09.019

